# High yield conversion of biowaste coffee grounds into hierarchical porous carbon for superior capacitive energy storage

**DOI:** 10.1038/s41598-020-60625-y

**Published:** 2020-02-26

**Authors:** Xiaoguang Liu, Shuai Zhang, Xin Wen, Xuecheng Chen, Yanliang Wen, Xiaoze Shi, Ewa Mijowska

**Affiliations:** Nanomaterials Physicochemistry Department, Faculty of Chemical Technology and Engineering, West Pomeranian University of Technology Szczecin, al. Piastów 45, 70-311 Szczecin, Poland

**Keywords:** Energy storage, Nanoscale materials, Materials for energy and catalysis, Nanoscale materials, Natural hazards, Sustainability

## Abstract

Recently great efforts have been focused on converting biowastes into high-valued carbon materials. However, it is still a great challenge to achieve high carbon yield and controllable porous distribution in both industrial and academic research. Inspired by the multi-void structure of waste coffee grounds, herein we fabricated hierarchical porous carbon via the combination of catalytic carbonization and alkali activation. The catalytic carbonization process was applied to obtain well-defined mesoporous carbon with carbon yield as high as 42.5 wt%, and subsequent alkali activation process produced hierarchical porous carbon with ultrahigh specific surface area (3549 m^2^ g^−1^) and large meso-/macropores volume (1.64 cm^3^ g^−1^). In three-electrode system, the electrode exhibited a high capacitance of 440 F g^−1^ at 0.5 A g^−1^ in 6 M KOH aqueous electrolyte, superior to that of many reported biomass-derived porous carbons. In two-electrode system, its energy density reached to 101 Wh kg^−1^ at the power density of 900 W kg^−1^ in 1-Ethyl-3-Methylimidazolium Tetrafluoroborate (EMIMBF_4_). This work provided a cost-effective strategy to recycle biowastes into hierarchical porous carbon with high yield for high-performance energy storage application.

## Introduction

In recent years, with the purpose of turning waste into treasure and reducing environmental hazard, waste management for the production of high-valuable carbon materials has received considerable attention^1^. Specially, biowastes are viewed as the most outstanding natural carbon precursors by virtue of their rich source, low-cost and sustainability^2–4^. So far, many biowastes have been recycled to synthesize carbon materials, but their applications are still limited by low carbon yield and unsatisfied pore structure^5–7^. Thus, effective methods are necessary to control carbonization process and optimize pore distribution for meeting the demands of various intended applications.

Until now, several strategies have been developed for the fabrication of porous carbons, including hydrothermal carbonization^8^, hard template^9^ and molten-salt route^10^, etc. Generally the widely used carbon-rich precursors are polymers^11^, metal-organic frameworks^12^, organic complex^13^ and carbohydrates^14^. Meanwhile, natural biomass is also widely used as carbon precursors, such as salvia splendens^15^, clover stems^16^, moringa oleifera branches^17^, ginkgo leaves^18^, and pollens^19^. Moreover, with considering environmental protection and resource recycle, biowastes are viewed as more promising natural carbon precursors^20^. Currently, various biowastes from plants or animals have been used for porous carbons production, such as kraft lignin^21^, soybean residue^22^, dairy manure^23^, bagasse wastes^24^ and peanut shell^1^. In many cases, the added catalysts (or activators) are difficult to penetrate into the inside of carbon precursors, resulting in low carbon yield and unmanageable pore distribution^25^. Besides, many kinds of biowastes are difficult to collect or have a low production. It is therefore paramount to exploit low-cost and widely-distributed biowastes as carbon precursors, followed by effective catalytic-carbonization process and optimize pore size distribution for achieving high carbon yield and controllable porous structure.

As an important energy-storage device, supercapacitor has been attracting significant interest due to its intrinsic advantages of high power density, rapid charge-discharge rate and excellent cycling stability^26–28^. The principle of supercapacitor is a quick charge accumulation and release process in the interface of electrode-electrolyte, where the characters of electrode materials are decisive to the resultant electrochemistry performances^9,29^. Among various electrode materials, porous carbons have been the most promising candidates for supercapacitor application^30,31^. The ideal porous carbons should have large specific surface area for charge storage, hierarchical pores (micro-, meso- and macro-pores) for fast ion transport/diffusion and good wettability for promoting the pore access to electrolyte ions. In this case, for meeting the requirements of ideal electrode materials, it is extremely imperative to select biowastes with special structure (such as naturally porous) as carbon precursors, followed by scalable synthetic methods for achieving high carbon yield and controllable porous structure.

Coffee is one of the most widely consumed beverages and more than five million tons is produced annually. The residues of coffee grounds (CGs) are composed of cellulose, hemicellulose and lignin, which may cause environment pollution if it is not properly disposed^32^. Besides these common features of biomass wastes, waste CGs possess a large number of *in-situ* formed voids due to the dissolution of interior molecules (caffeine, tannins, polyphenols, etc.) during boiling process (Fig. S1a,b by SEM, in supporting information). These uniformly distributed voids can act as reservoirs to store catalyst, which provides the great potential for effective catalytic-carbonization. In our recent work^33^, waste CGs were successfully converted into functional carbon materials by one-step catalytic carbonization. Notably, the synthesized carbon possessed good graphitization degree, high surface area and mesopore-dominant structure. Considering above-mentioned characters of ideal carbons, if the porosity of waste coffee grounds is further optimized into hierarchical porous architecture, the obtained carbon will be a promising material for high-performance supercapacitive electrodes.

**Figure 1:**
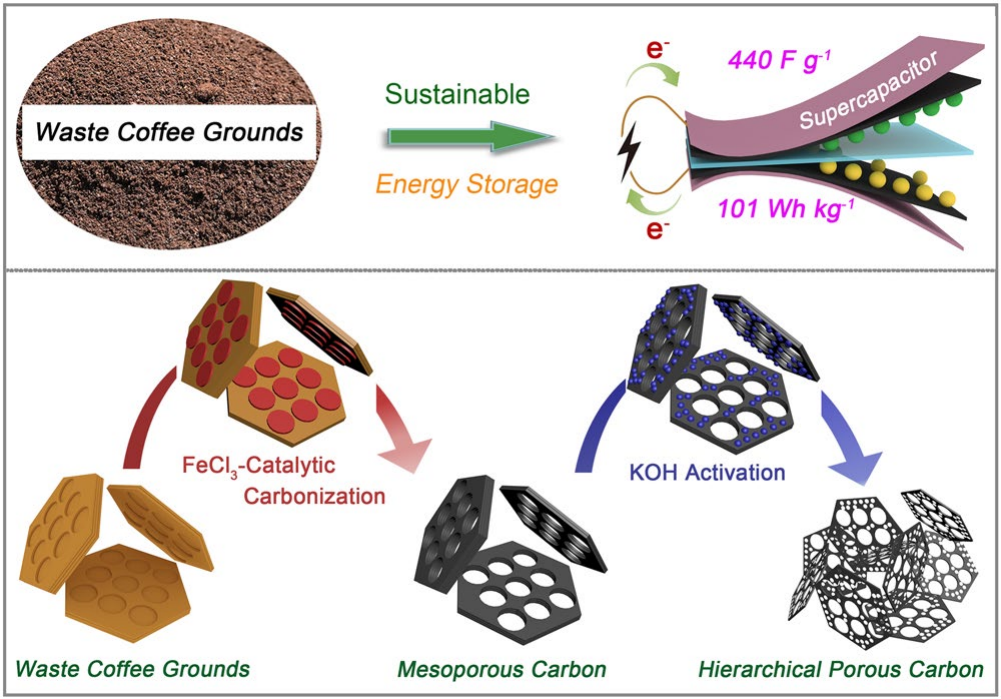
Synthesis process of hierarchical porous carbon from waste coffee grounds and its supercapacitor application.

In the present study, a combined strategy consisting of catalytic carbonization and alkali activation was developed to convert biowastes into hierarchical porous carbon. As shown in Fig. 1, waste CGs were firstly catalyzed into mesoporous carbon by FeCl_3_, then the micropore-created process was performed by KOH activation. Furthermore, the as-prepared hierarchical porous carbon was employed as supercapacitive electrodes, which exhibited a high capacitance of 440 F g^−1^ at 0.5 A g^−1^ using 6 M KOH electrolytes in three-electrode system. The fabricated symmetric supercapacitor also displayed good rate capability (81% capacitance retention at 20 A g^−1^) in two-electrode system. Meanwhile, the energy density as high as 101 Wh kg^−1^ at the power density of 900 W kg^−1^ was demonstrated in 1-ethyl-3-methylimidazolium tetrafluoroborate (EMIMBF_4_) electrolyte. We believe that it will not only open an effective route to recycle biowastes in large scale with high yield, but also develop a promising method to fabricate high-valuable carbon as electrodes for high-performance supercapacitors.

## Experimental Section

### Materials and preparation

Waste coffee grounds (CGs) were collected from coffee leftovers after boiling in a coffeemaker. The wet powders were dried in a vacuum oven under 80 °C for 6 h, and then they were crushed into tiny pieces (100 mesh). Ferric trichlorides (FeCl_3_), potassium hydroxide (KOH) and 1-Ethyl-3-Methylimidazolium Tetrafluoroborate (EMIMBF_4_) were purchased from Sigma-Aldrich Company, Ltd (Poland), and used directly without further purification.

Typically, CGs (3.0 g) was mixed with 0.6 g of FeCl_3_ in 30 ml deionized water and sonicated for 30 min. To achieve uniform distribution of catalyst, the slurry was frozen in a refrigerator followed by freeze-drying for 72 h. Subsequently the fluffy sample was pyrolyzed at 700 °C (10 °C min^−1^) in argon for 2 h using a tube furnace. The resulting carbonaceous solid was hydrothermal treated by 10 wt % hydrochloric acid solution at 120 °C for 6 h and washed with distilled water, then recovered by filtration and drying (labeled as biochar). The carbon yield in carbonization process of waste coffee grounds was 42.5 wt%, which was calculated by m_biochar_/m_waste coffee grounds_ × 100%, where the m_biochar_ is the weight of purified biochar (1.274 g) and the m_waste coffee grounds_ is the weight of waste coffee grounds (3.0 g). Subsequently, part of the obtained biochar (0.3 g) was thoroughly mixed with different amount of KOH (0.6, 1.2, or 1.8 g) in a mortar, then the mixture was put into a nickel crucible and heated at 800 °C (5 °C min^−1^) for 2 h under argon in the tube furnace. After cooling to room temperature, samples were purified using 10 wt % hydrochloric acid solution and distilled water, and dried in an oven at 100 °C for 12 h. According to the mass ratio of KOH/biochar = 2/1, 4/1 and 6/1, the obtained hierarchical porous carbons were named as HPC-2, HPC-4 and HPC-6. As a comparison, the biochar was also treated with the same process without additive of KOH, and the obtained meso-/macropores dominant carbon was labeled as MC. The carbon yield was calculated by the formula: m_carbon_/m_waste coffee grounds_ × 100%, where m_carbon_ is the weight of purified carbon after activation process.

In addition, a control experiment from directly carbonization of waste coffee grounds and followed by KOH activation were conducted. In short, the waste coffee grounds were directly carbonized at 700 °C (10 °C min^−1^) for 2 h in argon followed by purification using 10 wt% HCl solution and distilled water. The obtained biochar was mixed with KOH using mass ratio m_KOH_/m_biochar_ (4:1) and activated at 800 °C (5 °C min^−1^) for 2 h. After removal of impurities and drying, the product was collected and named as C-700-800.

### Materials characterization

The thermal stability of samples was investigated by thermogravimetric analysis (TGA) using a TA Instruments (DTA-Q600 SDT) at a heating rate of 10 °C min^−1^ from room temperature to 800 °C under air. Field-emission scanning electron microscopy (FE-SEM, JEOLJEM-1011) at 100 kV was applied to observe the morphology of samples. The microstructure of the samples was investigated by transmission electron microscopy (TEM) on FEI Tecnai F30 transmission electron microscope operating at an acceleration voltage of 200 kV. The vibrational properties of samples were measured by Raman scattering using a Renishaw-micro Raman spectrometer (λ= 532 nm). The surface element composition of samples was characterized by means of X-ray photoelectron spectroscopy (XPS) (VGESCALAB MKII spectrometer using an Al Kα exciting radiation from an X-ray source operated at 10.0 kV and 10 mA). The survey scanning mode was carried out to obtain the low-resolution spectra (0–1200 eV) while the XPSPEAK 4.1 software was applied to further deconvolute the high-resolution C1s spectra (282–292 eV) into four carbon-related Gaussian peaks. The textural properties of samples were characterized by N_2_ adsorption/desorption at liquid nitrogen temperature (77 K) using a Quantachrome Autosorb-1C-MS instrument. The multipoint-BET method (relative pressure range: 0.05 < P/P_0_ < 0.28) was applied to calculate the specific surface area. The total pore volume was determined at relative pressure P/P_0_ = 0.99. The Nonlocal Density Functional Theory (NLDFT, slit/cylinder pores) methods were used to calculate the pore volume and pore size distribution.

### Electrochemical measurements

The working electrode was prepared by firstly mixing the as-synthesized carbon materials, acetylene black and polytetrafluoroethylene (PTFE) using a mass ratio of 80/10/10 in ethanol, then the slurry was coated on a round nickel foam (D = 1.0 cm) and pressed at a pressure of 10 MPa for 30 s to form working electrode followed by drying at 100 °C for 12 h in an oven. The mass loading of active material was 4.0 mg and the surface loading was 5.1 mg cm^−2^. 6 M KOH aqueous solution or 1-Ethyl-3-Methylimidazolium Tetrafluoroborate (EMIMBF_4_) was selected as electrolytes. In three-electrode system, the Hg/HgO electrode and Pt plate were used as reference electrode and counter electrode, respectively. In two-electrode system, the symmetrical supercapacitor was assembled using two electrodes with a glassy fiber paper as separator. The slurry of the mixture (carbon materials/acetylene black/PTFE) was pasted on the aluminum foil in electrochemical test using EMIMBF_4_ electrolyte. The electrochemical performance was evaluated by cyclic voltammetry (CV), galvanostatic charge/discharge (GCD) and electrochemical impedance spectroscopy (EIS) measurements using an EC-LAB VMP3 potentiostat (BioLogic Science Instruments).

In three-electrode system, the specific capacitance (C, F g^−1^) of the electrode from CV curves and GCD profiles is calculated via the following equation:

1 $${C}_{electrode}=\frac{1}{2\,ms({V}_{b}-{V}_{a})}{\int }_{{V}_{a}}^{{V}_{b}}\,IdV$$

2 $${C}_{electrode}=I\ast \Delta t/(m\ast \Delta V)$$

where *I* (mA) is the current response, *V*_a_ and *V*_b_ are the upper limit and lower limit of potential, *s* (mV s^−1^) is the potential change per second, *∆t* (s) is the discharge time, *m* (mg) is the mass loading of active material on working electrode and *∆V* is the potential window (V) for GCD test.

The diffusion coefficient of supercapacitor is calculated using EIS data according to:

3 $${\rm{D}}={R}^{2}{T}^{2}/2{A}^{2}{n}^{4}{C}^{2}{F}^{4}{\sigma }^{2}$$

4 $$Z{\prime} ={R}_{ct}+{R}_{s}+\sigma {\omega }^{-1/2}$$

where ${\rm{D}},{\rm{R}},{\rm{T}},A,n,C,F,\sigma ,Z{\prime} ,{R}_{ct},{R}_{s}$ and $\omega$ are the diffusion coefficient, gas constant, absolute temperature, surface area, number of electrons, concentration of ions, Faraday’s constant, Warburg factor, real part of impedance, charge transfer resistance, spreading resistance and frequency, respectively.

In two-electrode system, specific capacitance (*C*, F g^−1^) of the electrode calculated from CV curves and GCD profiles, energy density (*E*, Wh kg^−1^) and power density (P, W kg^−1^) of the supercapacitor derived from GCD profiles are calculated via the following equation:

5 $${C}_{electrode}=\frac{1}{ms\,({V}_{b}-{V}_{a})}{\int }_{{V}_{a}}^{{V}_{b}}I\,dV$$

6 $${C}_{electrode}=2\times I\times {\Delta }t/(m\times {\Delta }V)$$

7 $$E=({C}_{electrode}\times {\Delta }{V}^{2})/(8\times 3.6)$$

8 $$P=(E\times 3600)/{\Delta }t$$

where *I* (mA) is the current response, *V*_a_ and *V*_b_ are the upper limit and lower limit of voltage, *s* (mV s^−1^) is the voltage change per second, *Δt* (s) is the discharge time, *m* (mg) is the mass loading of active material in single electrode and *ΔV* is the voltage change during discharge.

## Results and Discussion

### Carbon yields from waste CGs by catalytic carbonization and alkali activation

Our previous work has reported the catalytic carbonization of waste CGs in a crucible in a muffle furnace under air atmosphere^33^. To eliminate the effect of residual oxygen in crucible and achieve the precise control of temperature, here the carbonization process was carried out in a tube furnace under argon. Similarly, 20 wt% FeCl_3_ was added during the carbonization process of waste CGs, which leads to the carbon yield as high as 42.5 wt%, at least ten percent higher than that of previous reports^5,7,34–36^. The high carbon yield was ascribed to the advantage of its *in-situ* formed voids for efficiently adsorbing FeCl_3_, which could form iron-containing particles under high temperature. It was reported that the iron-containing particles played a catalyst role during the carbonization process of biomass/polymer, providing a high carbon yield^37^. Subsequently, a KOH activation process (mass ratio of m_KOH_/m_biochar_ = 0, 2, 4 or 6) was employed to activate carbon samples, named as MC, HPC-2, HPC-4 and HPC-6, respectively. As a result, the final carbon yields calculated from waste CGs were 41.3 wt% (MC), 34.5 wt% (HPC-2), 29.5 wt% (HPC-4) and 22.3 wt% (HPC-6).

### Physical characterization of waste CGs derived carbons

**Figure 2:**
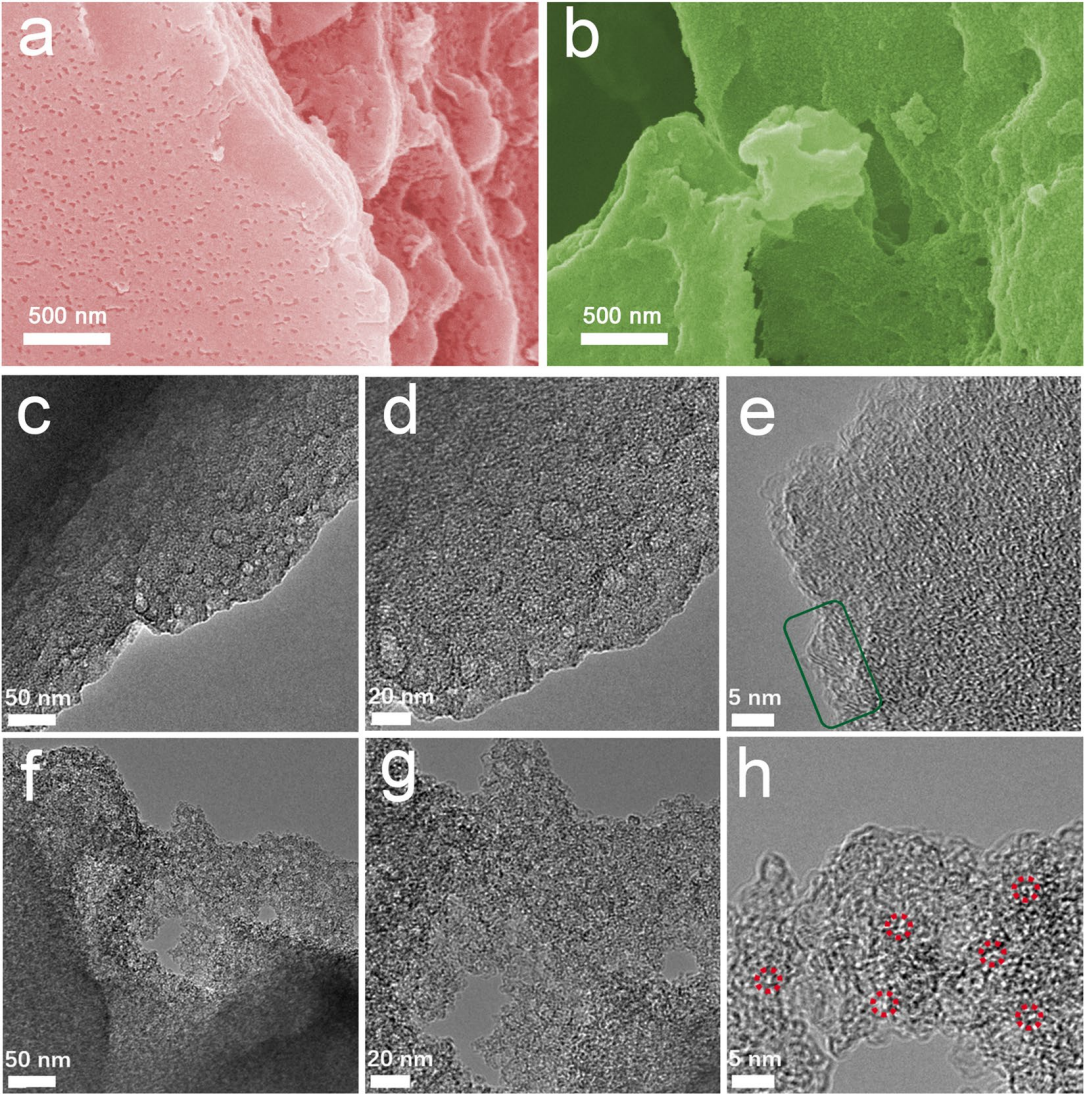
SEM images of waste CGs derived carbons: (**a**) MC and (**b**) HPC-4; TEM images of (**c**–**e**) MC and (**f**–**h**) HPC-4 (green box indicates the lattice fringe in MC and red circles represent the micro-pores in HPC-4).

The morphology and microstructure of as-obtained carbons were characterized by SEM and TEM. A large amount of mesopores were homogeneously distributed on the surface of MC (Fig. 2a). Generally, the agglomeration of iron-containing particles under high temperature could act as template to form abundant mesopores in the resultant carbons. After activation, the HPC-4 possessed blanket-like rough surface, indicating the formation of abundant micropores (Fig. 2b). As shown in Fig. 2c,d, MC possesses abundant mesopores with various diameters from several nanometers to dozens nanometers. Interestingly, despite its mesoporous structure, the high-resolution TEM image of MC validates its partially graphitized structure with regular lattice fringe (green box in Fig. 2e). Fragmentized and thinner carbon sheets (Fig. 2f,g) as well as the abundant micropores (red circles in Fig. 2h) were confirmed by the TEM, revealing its amorphous character. These results demonstrated that the FeCl_3_ catalyst could create the mesopores-dominant structure and the subsequent KOH activation further introduced abundant micropores to form the hierarchical porous structure.

**Figure 3:**
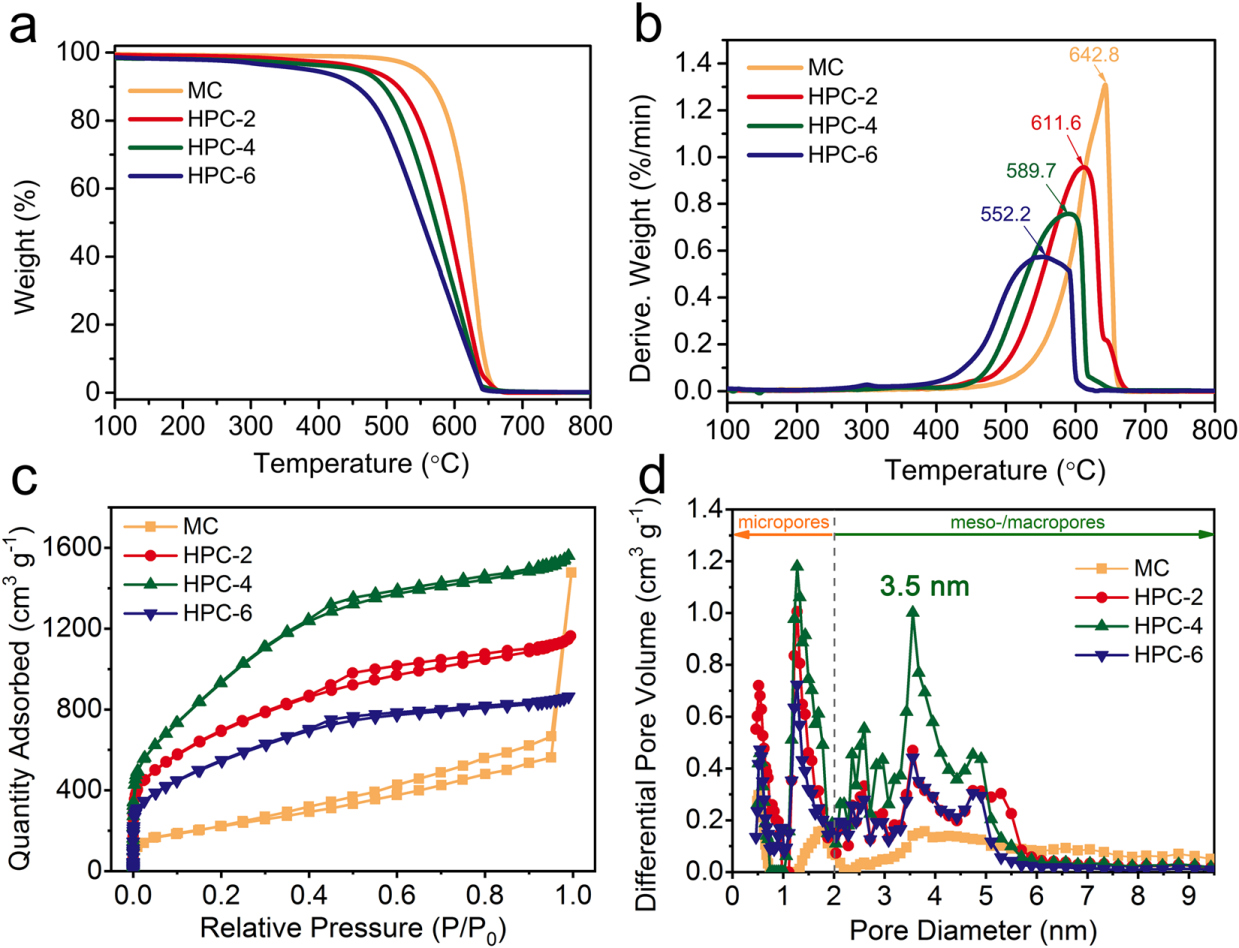
(**a**) TGA, (**b**) DTG curves, (**c**) Nitrogen adsorption/desorption isotherms and (**d**) pore size distribution of waste CGs derived carbons calculated by NLDFT method.

The graphitic nature and purity of waste CGs derived carbons were investigated by TGA under air atmosphere. As shown in Fig. 3a, the residues for all samples at 800 °C was very close to zero, indicating that the derived products from metal catalyst and KOH were completely removed after purification. It is well-known that iron-containing compounds (Fe_3_O_4_, Fe_3_C, etc.) easily grew inside carbon matrix under high temperature^38^, therefore hydrothermal treatment was carried out to remove iron species completely and obtain high-purified carbon. Figure 3b shows DTG curves of carbon samples. With the increase of KOH amount, the maximum oxidation temperature (*T*_max_) gradually shifted to low temperature region (642.8 °C for MC and 552.2 °C for HPC-6), and the *T*_max_ peak became much broader, suggesting that the etching effect of alkali destroyed the ordered graphitized structure and introduced relatively more defects (pores, functional groups, etc.) into the carbon skeleton^39^. In Raman spectra (Fig. S2), the peak centered at 1352 cm^−1^ (D-band) was a reflection of the A_1g_ vibration mode in defective carbon structure, while another peak located at 1585 cm^−1^ (G-band) was assigned to the stretching bond in sp^2^ hybridized carbon^40^. The intensity ratio of the G and D peak is used to evaluate the quality of obtained carbon materials^41^. Significantly, the integrated intensity ratio (*I*_G_/*I*_D_) was determined to be 1.07, 0.98, 0.97 and 0.94 for MC, HPC-2, HPC-4 and HPC-6 respectively, verifying that more defects were introduced via KOH treatment.

The porosity of waste CGs derived carbons was analyzed by N_2_ adsorption−desorption measurements. As depicted in Fig. 3c, the weaken gas adsorption within low relative pressure and a steep gas adsorption within higher relative pressure for MC belongs to typical type IV isotherms with H3 hysteresis, demonstrating the existence of less micropores and dominance of meso-/macropores. This result further verified the significant role of FeCl_3_ that mainly introduced meso-/macropores into carbon matrix during carbonization, as consistent with the TEM result. Alkali activation is widely used to produce micropores-dominant carbon with large specific surface area (SSA) and abundant micropores, as confirmed by previous reports^42,43^. In our work, HPCs displayed a typical I/IV pattern with a H4 hysteresis loop after KOH activation process, indicating the presence of abundant micropores and meso-/macropores. The pore size distribution of these carbons was shown in Fig. 3d, revealing that the mesopores of HPCs mainly centered at 3.5 nm. Compared with the MC, the HPCs have more micropores (1–2 nm). Furthermore, the S_BET_ increased from 801 m^2^ g^−1^ for MC to 3549 m^2^ g^−1^ for HPC-4, revealing the strong pore-making ability of KOH. There was an anomalous reduced S_BET_ for HPC-6, which was probably caused by the excessive KOH etching that resulted in the structural collapse of pores (Table 1). Among the HPCs samples, the HPC-4 possessed the highest S_BET_ (3549 m^2^ g^−1^), the biggest micropore volume (V_micropore_ = 0.64 cm^3^ g^−1^) and wide pore size distribution with adequate mesopores (V_meso-/macropores_ = 1.64 cm^3^ g^−1^). These unique characters will enhance the ions storage capacity and minimize the ions diffusion limitation, thereby endows it an ideal electrode material for supercapacitor with promising electrochemical performance. As a reference, the C-700–800 exhibited a smaller S_BET_ of 1218 m^2^ g^−1^ and lower meso-/macropores volume of 0.11 cm^3^ g^−1^ (Table S1). These results indicated that the catalytic carbonization process of FeCl_3_ played a significant role to create abundant meso-/macropores.

**Table 1:** N_2_ adsorption–desorption isotherm parameters for waste CGs derived carbons.

Samples	S_BET_ (m^2^ g^−1^)	V_t_ (cm^3^ g^−1^)	V_t-DFT_ (cm^3^ g^−1^)	V_micropores-DFT_ (cm^3^ g^−1^)	V_meso/macropores-DFT_ (cm^3^ g^−1^)
MC	801	1.03^a^	1.01	0.11	0.90
HPC-2	2513	1.80	1.69	0.58	1.11
HPC-4	3549	2.41	2.28	0.64	1.64
HPC-6	1999	1.34	1.27	0.42	0.85

^a^The total pore volume of MC is determined at P/P_0_ = 0.95 to avoid the problem of nitrogen condensation.

**Figure 4:**
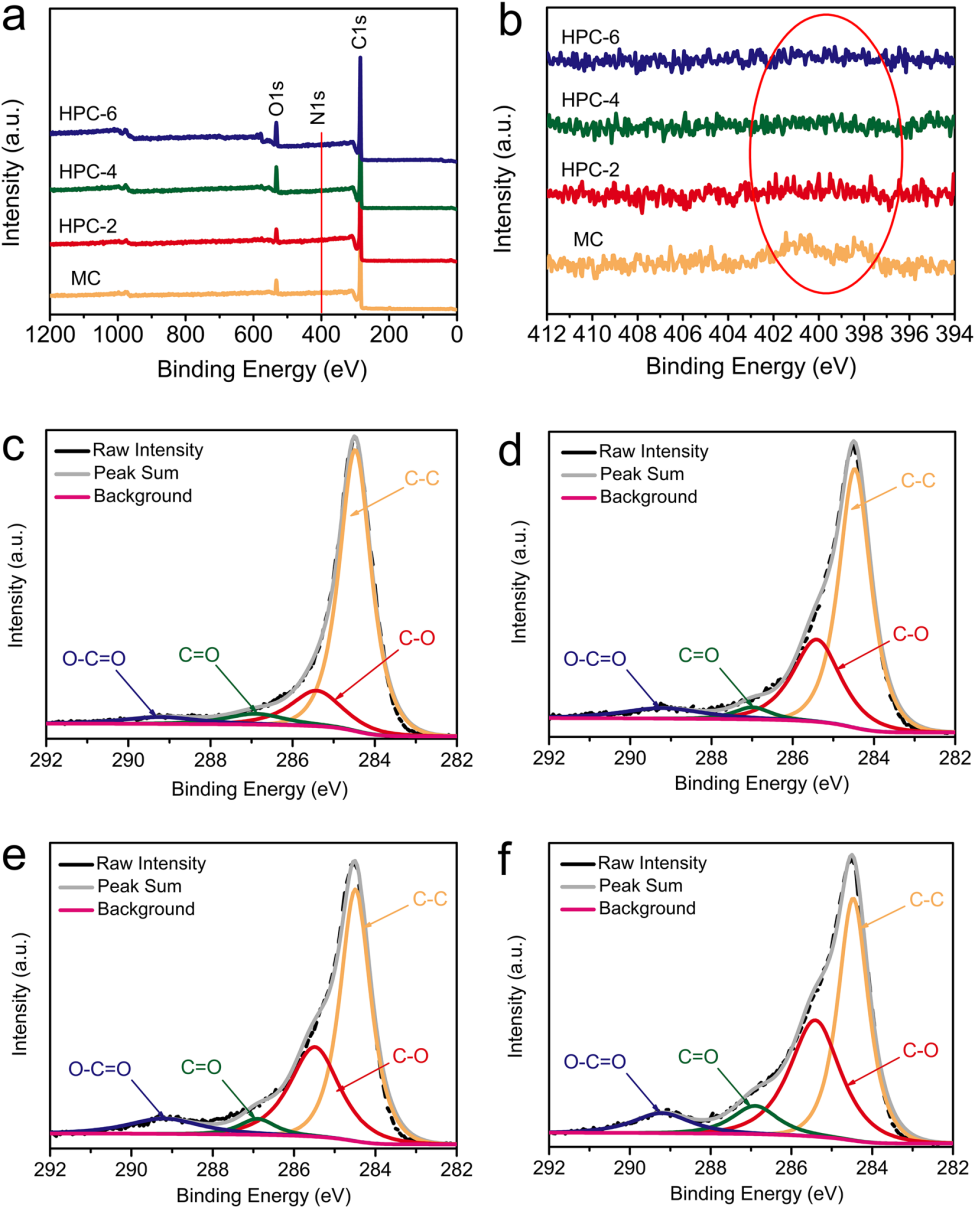
(**a**)XPS survey and (**b**) N1s spectra of samples; high-resolution spectra of C1s for (**c**) MC, (**d)** HPC-2, (**e**) HPC-4 and (**f**) HPC-6.

The element composition of carbon samples was characterized by XPS. As shown in Fig. 4a,b, the survey spectra confirmed that the MC contains abundant carbon, oxygen and a little nitrogen (1.2%). The oxygen content increases from 5.6% for MC to 11.9% for HPC-6, while the nitrogen element became undetectable for HPCs. Moreover, the high-resolution C1s XPS spectra can be fitted into four individual peaks: C−C (284.6 eV), C−O (285.8 eV), C=O (287.1 eV) and O−C=O (288.9 eV) (Fig. 4c–f). It is clearly observed that the C−O and O−C=O content significantly increased during activation process (The detailed data were summarized in Table S2 in supporting information). It was reported that the incorporation of oxygen-containing groups into the carbon matrix could not only effectively enhance the capacitance by providing the pseudocapacitance, but also improve the wettability of fabricated electrode^44^. Therefore, the abundant oxygen-containing groups in our carbon materials may play an important role in their electrochemical performance.

### Electrochemical performance evolution in three-electrode system

**Figure 5:**
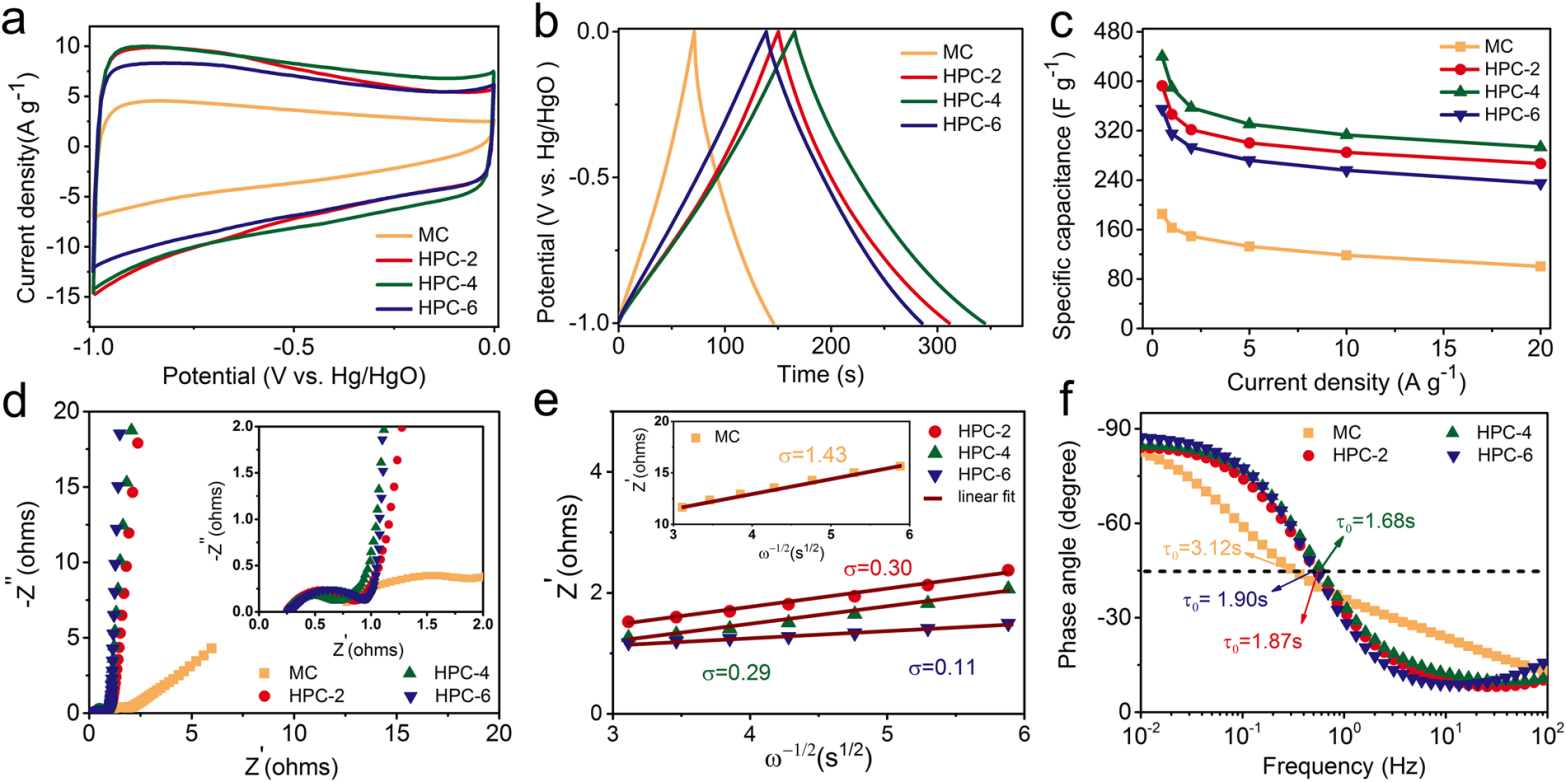
Electrochemical performance of waste CGs derived carbons in a three-electrode system using 6 M KOH electrolyte: (**a**) CV curves at 20 mV s^−1^; (**b**) GCD profiles at 2 A g^−1^; (**c**) Comparison of the capacitance at different current densities; (**d**) Nyquist plots; (**e**) A plot of real impedance as a function of ω^−1/2^ and (**f**) Bode phase plots.

The electrochemical properties of coffee grounds derived carbons were analyzed in a three-electrode configuration using 6.0 M KOH electrolyte. Figure 5a shows the CV curves at a scan rate of 20 mV s^−1^ with the potential range of −1 V to 0 V. It was apparent that the CV curve of HPC-4 displayed a quasi-rectangular shape without redox peaks, indicating the dominance of electric double-layer capacitive (EDLC) behavior during the charge-discharge process. Meanwhile, the highest discharge current and the biggest integral area of HPC-4 suggested its highest specific capacitance. The rectangular shape maintained even at a high scan rate of 200 mV s^−1^ (Fig. S3), revealing the important role of sufficient meso-/macropores in the carbon structure that mitigates the ions diffusion limitation. The maximum capacitance was 466 F g^−1^ at 1 mV s^−1^ and it dropped to 324 F g^−1^ at 200 mV s^−1^ (Fig. S4). As shown in Fig. 5b, the GCD profiles at 2 A g^−1^ displayed well-defined triangular shapes, which is indicative of typical capacitor behavior.

The calculated specific capacitances based on the discharge profiles at 0.5 A g^−1^ were 185, 393, 440 and 350 F g^−1^ for MC, HPC-2, HPC-4 and HPC-6, respectively. Notably, herein the capacitance of HPC-4 was much higher than 123 F g^−1^ of commercial activated carbon (YP-50F, Kuraray) tested under same condition (Fig. S5), and it was also superior to many other previously reported biomass-derived carbons (Table 2). In general, high-rate charge-discharge process usually results in the deterioration in charge storage capacity because of limited time for electrolyte ions diffusion within the whole electrode^45^. But in our work, with increasing the current density up to 10 A g^−1^, a high capacitance of 313 F g^−1^ was obtained (Fig. 5c). Even at a high current density of 20 A g^−1^, a superior rate capability (293 F g^−1^, 67% capacitance retention) is obtained, implying the fast ion diffusion rate. In addition, the reference sample (C-700-800) with less meso-/macropores exhibited a capacitance of 241 F g^−1^ at 0.5 A g^−1^ (Fig. S6a), and this value quickly dropped to 128 F g^−1^ at 20 A g^−1^, so its capacitance retention was only 53% (Fig. S6b). These results revealed the important role of sufficient meso-/macropores in fast ions transport for enhanced rate capability.

**Table 2:** Comparison of the electrochemical capacitance of biomass-derived carbon-based electrode in aqueous and organic electrolyte.

Biomass	Test system (electrode)^a^/Electrolyte	Current density (A g^−1^)	Specific capacitance (F g^−1^)	Energy density (Wh kg^−1^)	Power density (W kg^−1^)	Ref.
Bagasse	3E/6 M KOH	0.5	320	—	—	^24^
	2E/6 M KOH	0.2	180	6.3	100	
Cashmere	3E/6 M KOH	0.5	363	—	—	^48^
	2E/TEABF_4_	0.5	81	18	125	
Fungus	3E/6 M KOH	0.5	374	—	—	^49^
Silk	2E/EMIMBF_4_	1	213	90	875	^50^
Tofu	3E/6 M KOH	1	418	—	—	^10^
Artemia shells	3E/6 M KOH	0.5	426	—	—	^51^
Mushroom	3E/6 M KOH	1	306	—	—	^52^
	2E/6 M KOH	0.2	238	8.2	100	
	2E/TEABF_4_	0.5	149	31.7	620	
Protein	3E/1 M H_2_SO_4_	0.2	390	—	—	^53^
Seed dregs	3E/6 M KOH	1	333	—	—	^54^
Cinnamon	2E/NaClO_4_	0.5	225	70	375	^55^
Cherry calyces	2E/EMIMBF_4_	1	173	81.4	446.3	^56^
Coconut shell	2E/6 M KOH	1	276	9.6	500	^57^
Lignin	2E/EMIMBF_4_	0.1	192	64.2	74.5	^58^
Albizzia flower	3E/6 M KOH	0.5	406	—	—	^59^
Coffee grounds	3E/6 M KOH	0.5	440	—	—	**This work**
	2E/6 M KOH	0.5	319	11.1	125	
	2E/EMIMBF_4_	1	224	101	900	

^a^2E/3E indicates a two-electrode/three-electrode system.

Electrochemical impedance spectroscopy (EIS) measurement in the frequency range of 100 kHz to 10 mHz was applied to investigate the different ion transport/charge transfer properties. The Nyquist plots featured the semicircle in the high-frequency region followed by the 45 Warburg-type line and straight line in the low-frequency region, suggesting the combination of charge transfer and ion transport behavior in supercapacitor (Fig. 5d). In the high-frequency region, the smallest diameter of semicircle for HPC-4 marked its lowest R_ct_ with a fast charge transfer rate. Furthermore, the equivalent circuit model was employed to evaluate the R_ct_ values of fabricated supercapacitors, which was entirely consistent with Nyquist data to confirm the lowest R_ct_ (0.53 Ω) of HPC-4 (Fig. S7). In the low-frequency region, the straight line of HPC-4 was almost parallel to -Z′′ axis, revealing its typical double-layer capacitive behavior without ion diffusion limitation. Moreover, the diffusion coefficient (D, calculated from Eqs. (3) and (4) in electrochemical measurements part) was used to quantitatively compare the ion diffusion behavior in supercapacitors. As a key parameter reflecting the ion diffusion impedance, the Warburg factor (σ) can be obtained from the relationship between Z′ and ω^−1/2^ (Fig. 5e). The plots displayed linear features with different slopes (σ) of 1.43, 0.30, 0.29 and 0.11 for MC, HPC-2, HPC-4 and HPC-6, respectively. As a result, the calculated diffusion coefficient (D) for HPC-4 was about 24 times higher than that of MC.

By extrapolating the vertical part of the plot to the real axis, the obtained intercept was named as equivalent series resistance (R_ESR_), which represents the combination of ohmic resistance of electrolyte, electrical resistance of electrode and ionic diffusion resistance in the electrode^46^. A low R_ESR_ of 1.03 Ω was obtained for HPC-4 (Fig. S8). Furthermore, the Bode plots showed that the phase angles of all samples were close to −90° at 0.01 Hz, suggesting their ideal capacitive behavior (Fig. 5f). The characteristic frequency (f_0_) at −45° marks the point at which the capacitive impedance equals to resistive impedance. The f_0_ was 0.32 Hz, 0.53 Hz, 0.59 Hz and 0.53 Hz for MC, HPC-2, HPC-4 and HPC-6, respectively. The derived time constant t_0_ (t_0_ = 1/f_0_) was calculated to be as short as 1.68 s for HPC-4, much shorter than 10 s of commercial activated carbon-based supercapacitors. The rapid frequency response of HPC-4 further confirmed its better ion transport behavior that relied on the large ion-accessible SSA and the hierarchical porous structure.

### Electrochemical performance evolution in two-electrode system

To evaluate its practical application in energy storage, HPC-4 was assembled in a two-electrode symmetric supercapacitor using 6 M KOH electrolyte. The rectangular shape of CV curves maintained at a scan rate as high as 200 mV s^−1^, indicating the key role of hierarchical pores that facilitated fast ions transport throughout the entire electrode during short time (Fig. S9a). The GCD profiles exhibited the well-defined triangle shape at various current densities (Fig. S9b). The specific capacitance was 319 F g^−1^ at 0.5 A g^−1^ for HPC-4 based electrode. Furthermore, at a high current density of 20 A g^−1^, a high capacitance of 259 F g^−1^ was still remained with a good rate capability of 81% (Fig. S9c). Meanwhile, a high coulombic efficiency (>96%) was achieved. In addition, the long cycle test exhibited 94% capacitance retention after 10000 cycles at a current density of 10 A g^−1^ for HPC-4 based supercapacitor (Fig. S9d). The Nyquist plots shifted to right position after long cycling test, indicating the slightly increased equivalent series resistance.

**Figure 6:**
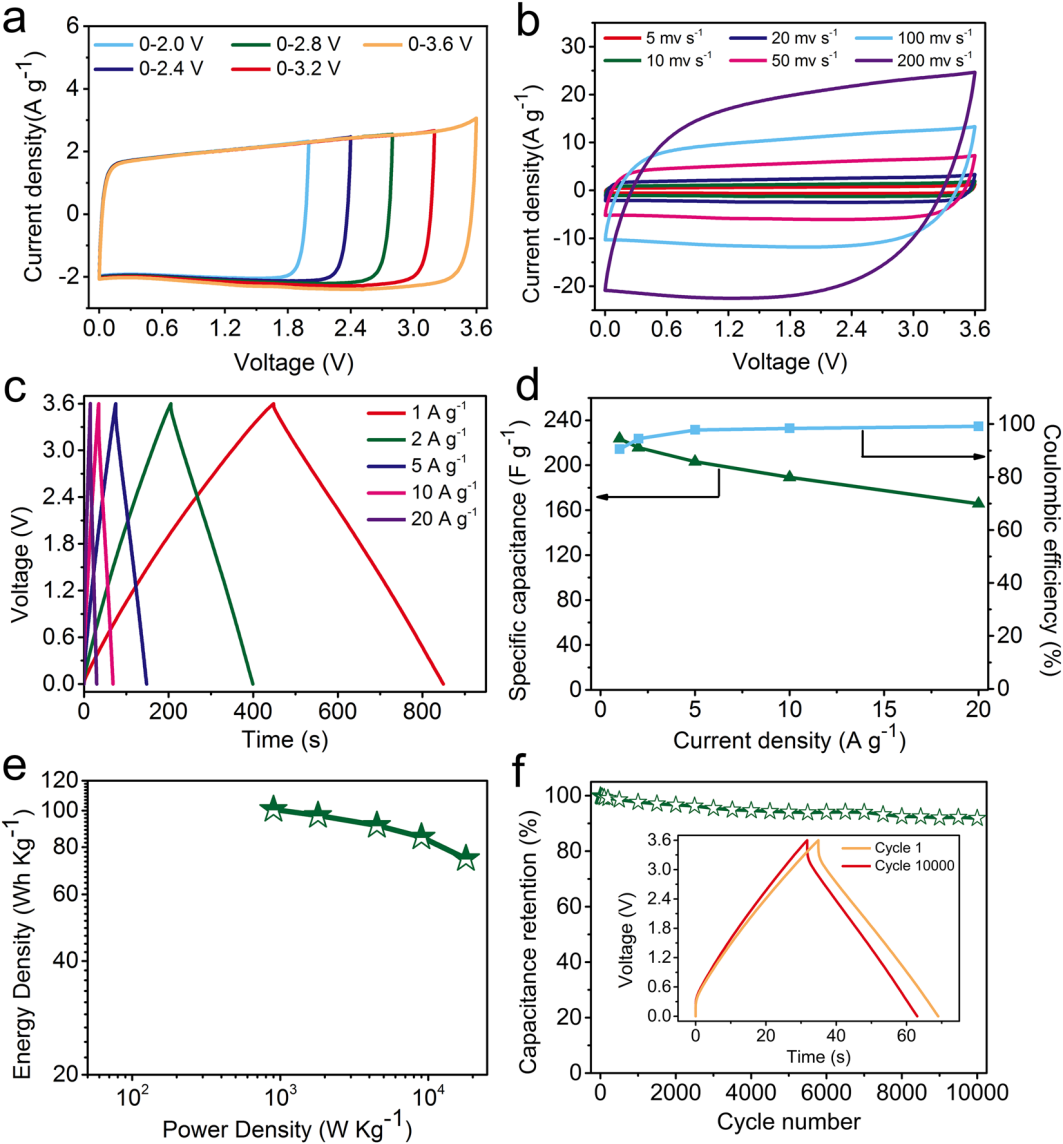
Electrochemical performance of HPC-4 in neat EMIMBF_4_: (**a**) CV curves at 20 mV s^−1^ at different operation voltages; (**b**) CV curves from 5 to 200 mV s^−1^; (**c**) GCD profiles from 1 to 20 A g^−1^; (**d**) Capacitance and coulombic efficiency as a function of the current density; (**e**) Ragone plot; (**f**) Cycle stability of HPC-4 at 10 A g^−1^.

The electrochemical properties of HPC-4 were further investigated in ionic liquid due to its wide electrochemical window as compared to aqueous electrolytes. Two symmetric electrodes were assembled in coin-type cell using neat EMIMBF_4_ electrolyte. Initially the CV measurement at a scan rate of 20 mV s^−1^ was carried out in voltage ranging from 0–2.0 V to 0–3.6 V to test the stability of fabricated device using neat EMIMBF_4_ (Fig. 6a). Previous work has revealed that the neat EMIMBF_4_ can be operated under the voltage window from 0–3 V to 0–4 V without decomposition^44,47^. At 0–3.6 V, our fabricated device exhibited the rectangular CV curve without apparent increase of anodic current, indicating its good EDLC behavior and superior reversibility. Therefore, the electrochemical performance was further studied based on CV curves and GCD profiles within a voltage window of 0–3.6 V.

Similar to the behavior in 6 M KOH electrolyte, even at a high scan rate of 200 mV s^−1^, CV curve still maintained a quasi-rectangular shape (Fig. 6b). The maximum capacitance of HPC-4 based electrode calculated from CV curves is 254 F g^−1^ at 5 mV s^−1^ (Fig. S10). Meanwhile, HPC-4 exhibited a symmetric triangular shape at different current densities from 1 to 20 A g^−1^ (Fig. 6c). The calculated specific capacitance of HPC-4 based electrode was 224 F g^−1^ at 1 A g^−1^, which was much better than that of most biomass-derived porous carbons (Table 2). Increasing the current density up to 20 A g^−1^, the high capacitance of 166 F g^−1^ was still present (Fig. 6d). It was believed that the high capacitance and good rate capability were ascribed to the abundant meso-/macropores that guarantees rapid diffusion of larger EMIM^+^ ions to access the inner surfaces. Meanwhile, the device showed a high coulombic efficiency of 91% at 1 A g^−1^. Moreover, the obtained Nyquist plot showed a straight line parallel to -Z′′ axis, which indicates the good electrolyte ions transport/charge transfer capacity (Fig. S11). In addition, the specific energy density (E, Wh kg^−1^) and power density (P, W kg^−1^) of HPC-4 based supercapacitor was calculated based on Eqs. (7) and (8). As our expected, the HPC-4 exhibited a high energy density of 101 Wh kg^−1^ at power density of 900 W kg^−1^, and still remained 75 Wh kg^−1^ at a power density of 18 kW kg^−1^ (Fig. 6e). These values were at least comparable or higher than that of most other biomass derived carbon materials (Table 2). Besides, long cycle test demonstrated good stability with high capacitance retention of 92% over the 10000 charge–discharge cycles at a current density of 10 A g^−1^ (Fig. 6f).

## Conclusions

In summary, we developed a cost-effective combined strategy to fabricate hierarchical porous carbon from waste coffee grounds by catalytic carbonization and alkali activation, and explored it application for high performance supercapacitive electrodes. A well-defined mesoporous carbon was obtained with a high carbon yield of 42.5 wt% via catalytic carbonization process, while subsequent activation process produced hierarchical porous carbon, which consisted of 3D porous architecture with ultrahigh surface area (3549 m^2^ g^−1^) and large meso-/macropores volume of 1.64 cm^3^ g^−1^. These advantageous features were beneficial to maximize charge storage capacity and fast ions diffusion/transport rate. In three-electrode system, the HPC-4 based electrodes displayed high specific capacitance (440 F g^−1^ at 0.5 A g^−1^) in 6 M KOH aqueous electrolyte. In two-electrode system, the fabricated symmetric supercapacitor exhibited an outstanding rate capability (81% capacitance retention at 20 A g^−1^). Moreover, a maximum energy density of 101 Wh kg^−1^ was achieved at a power density of 900 W kg^−1^ in EMIMBF_4_ electrolyte, which was comparable or much higher than that of most other biomass derived carbon materials. We believe that this combined strategy will be applicable to recycle not only waste coffee grounds, but also other biowaste resources. It will open a door for fabricating promising carbon materials and developing more exciting applications in the field of energy storage.

## Supplementary information

Supplementary information.
