# Supplementary material for: High yield conversion of biowaste coffee grounds into hierarchical porous carbon for superior capacitive energy storage
Source: Sci Rep. 2020 Feb 26;10:3518. doi: 10.1038/s41598-020-60625-y (PMC7044333; doi:10.1038/s41598-020-60625-y)
Supplement: Supplementary file 1 — Supplementary information. [file 41598_2020_60625_MOESM1_ESM.pdf]

Supporting information for

High yield conversion of biowaste coffee grounds into hierarchical porous carbon for superior capacitive energy storage

Xiaoguang Liu, Shuai Zhang, Xin Wen*, Xuecheng Chen*, Yanliang Wen, Xiaoze Shi, Ewa Mijowska

Nanomaterials Physicochemistry Department, Faculty of Chemical Technology and Engineering, West Pomeranian University of Technology Szczecin, al. Piastów 45, 70-311,Szczecin, Poland

*Corresponding author. Tel: +48-914496030

E-mail: hgwenxin@126.com (X. Wen); xchen@zut.edu.pl (X. Chen)

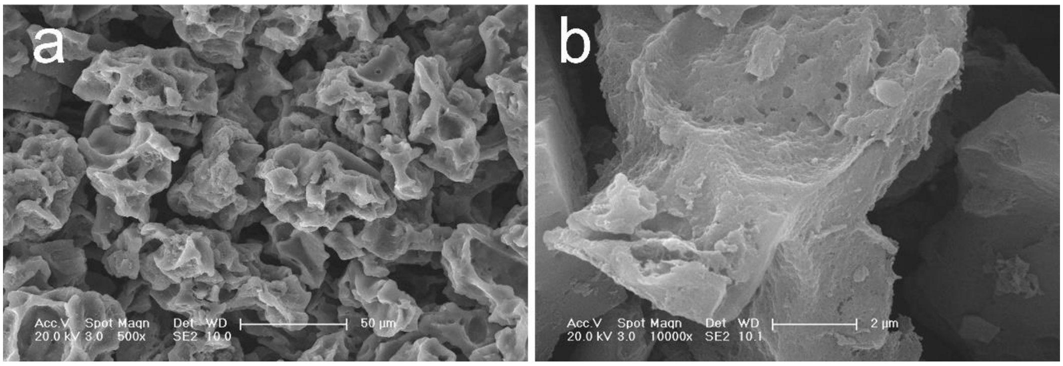

Figure S1. SEM images for waste coffee grounds in (a) low magnification (50 um) and (b) high magnification (2 um).

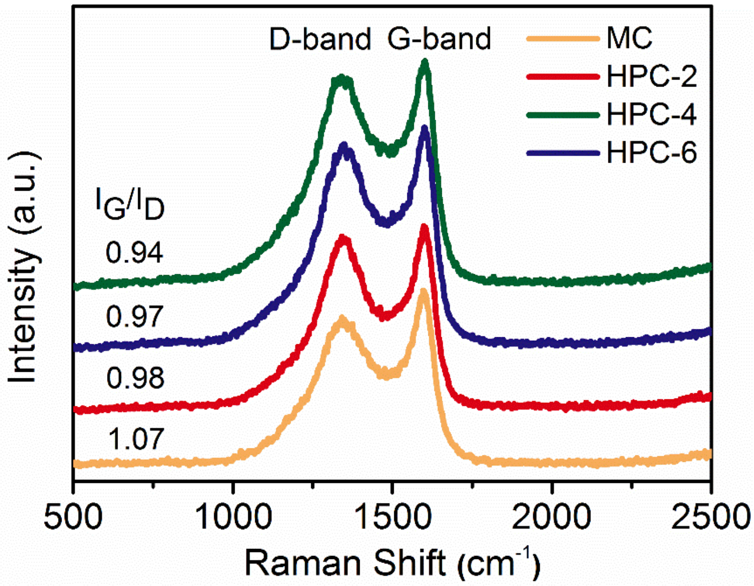

Figure S2. Raman spectroscopy of waste coffee grounds derived carbons.

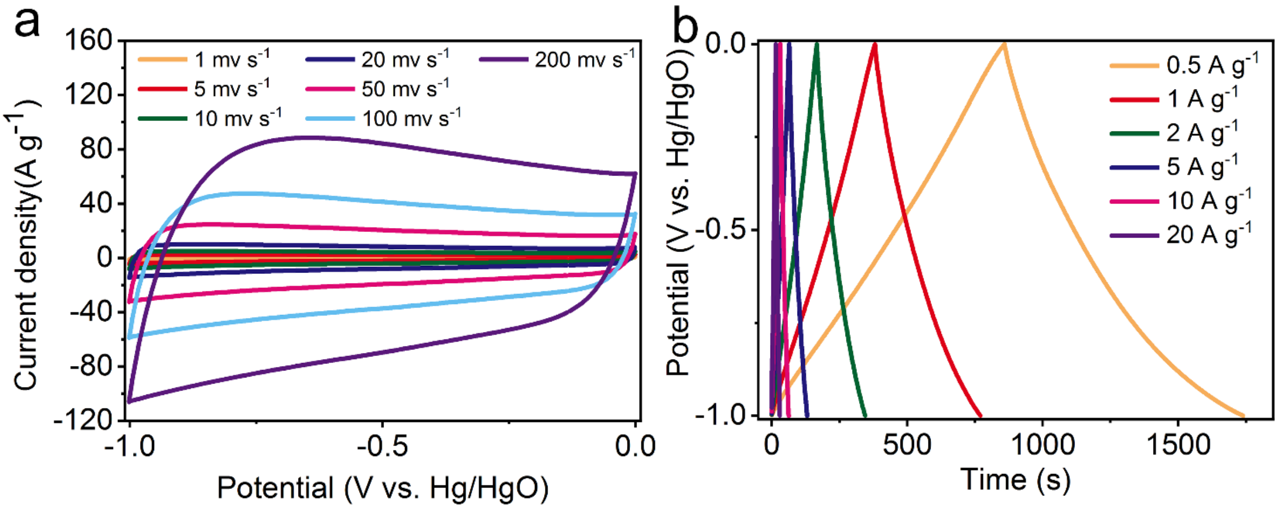

Figure S3. (a) CV curves and (b) GCD profiles of HPC-4 based electrode in three-electrode system in 6M KOH.

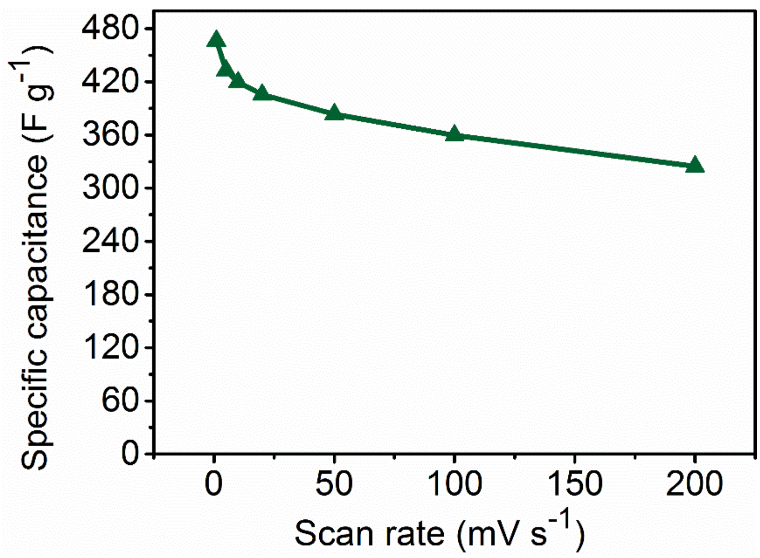

Figure S4. Specific capacitance calculated from CV curves for HPC-4 based electrode in three-electrode system in 6M KOH.

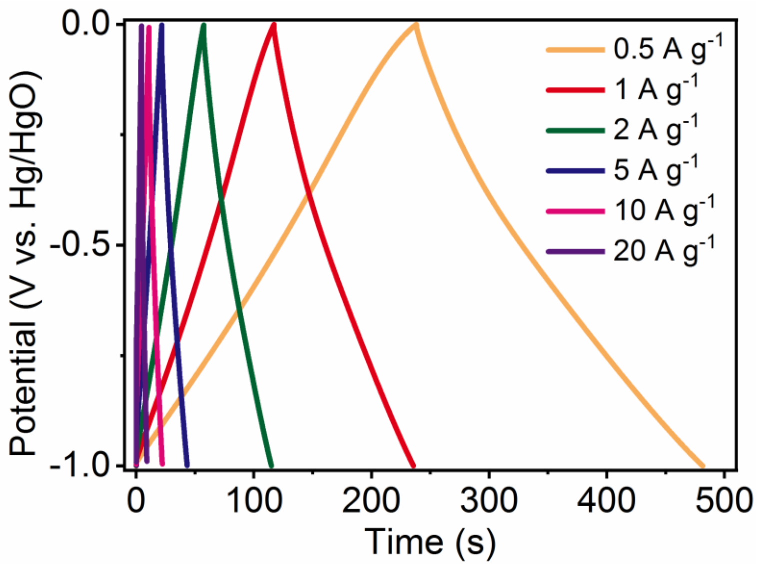

Figure S5. GCD profiles for commercial activated carbon (YP-50F) based electrode in three-electrode system in 6M KOH.

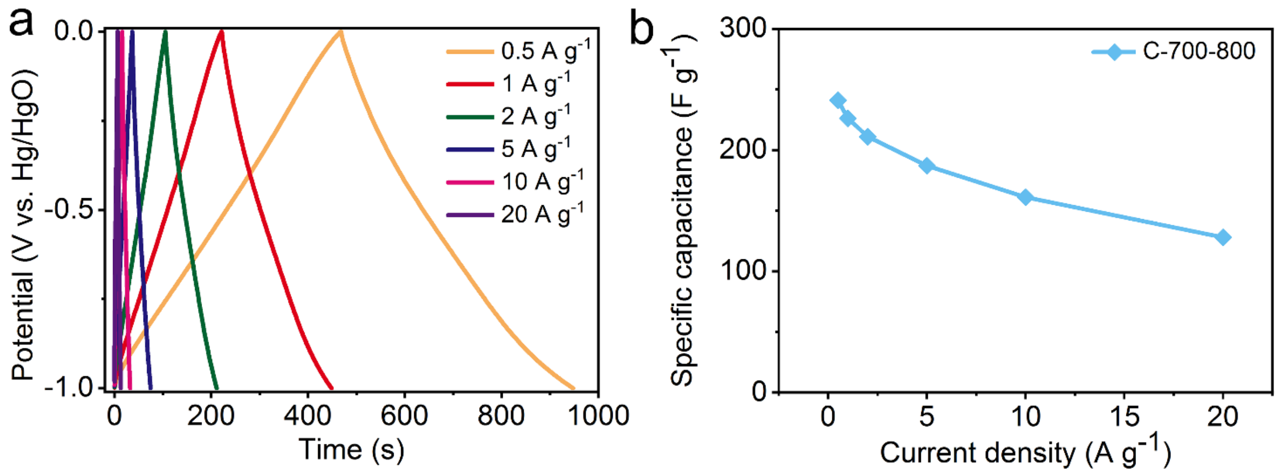

Figure S6. (a) GCD profiles for C-700-800 based electrode; (b) A plot of specific capacitance of C-700-800 based electrode as a function of current density in three-electrode system in 6M KOH.

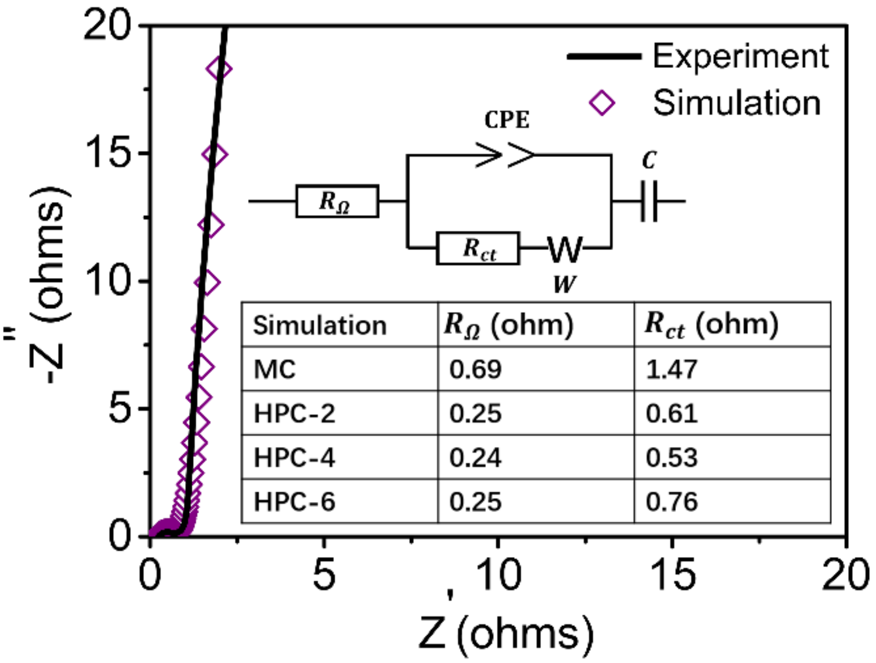

Simulation	R_Ω (ohm)	R_{ct} (ohm)
MC	0.69	1.47
HPC-2	0.25	0.61
HPC-4	0.24	0.53
HPC-6	0.25	0.76

Figure S7. Comparison of Nyquist plots in experiment and simulation of HPC-4 (insets are equivalent circuit diagram and ohm/charge transfer resistances of four simulate supercapacitors).

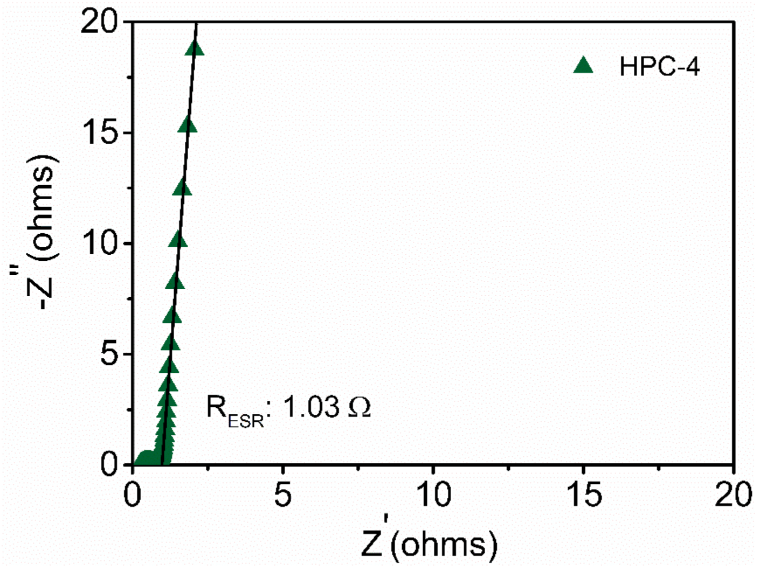

Figure S8. R_{ESR} of HPC-4 in three-electrode system in 6M KOH.

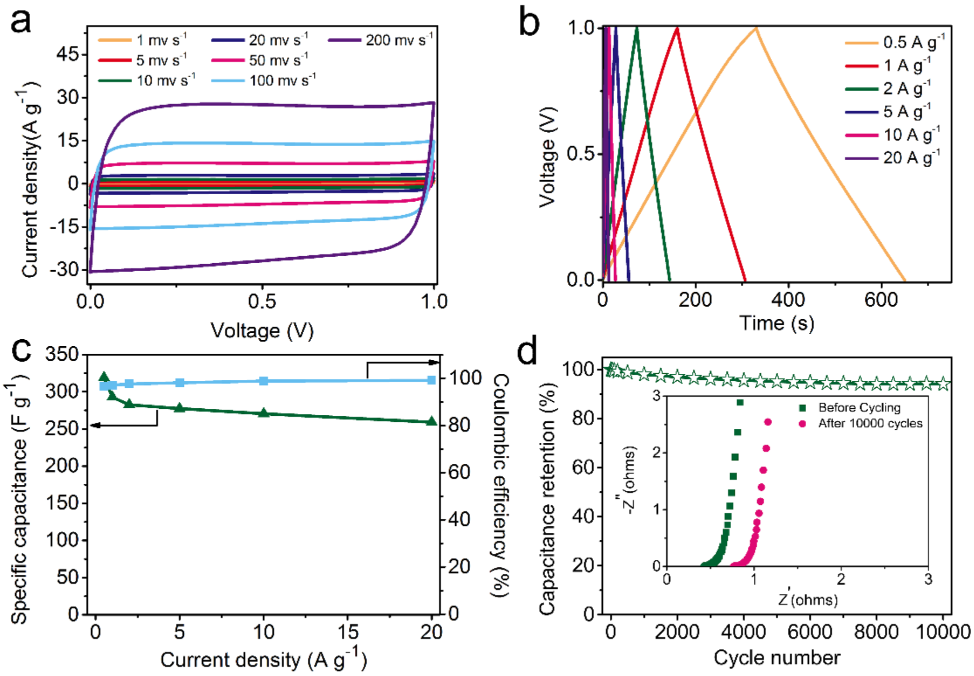

Figure S9. Electrochemical performance of HPC-4 based symmetric supercapacitor measured in two-electrode system using 6 M KOH electrolyte: (a) CV curves; (b) GCD profiles; (c) Comparison of specific capacitance and columbic efficiency versus various current densities; (d) Long cycle stability of HPC-4 at a current density of 10 A g^{-1} (inset displays the Nyquist plots before and after 10000 cycles).

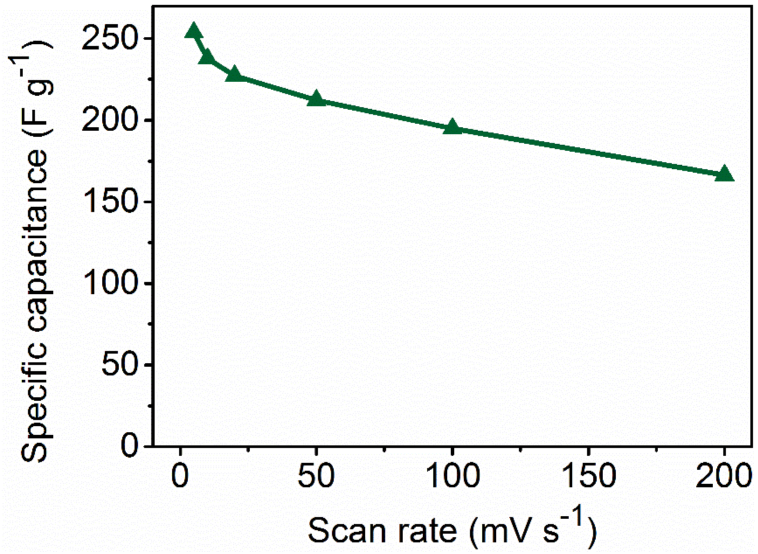

Figure S10. Specific capacitance calculated from CV curves for HPC-4 based electrode in two-electrode system in neat $EMIMBF_4$.

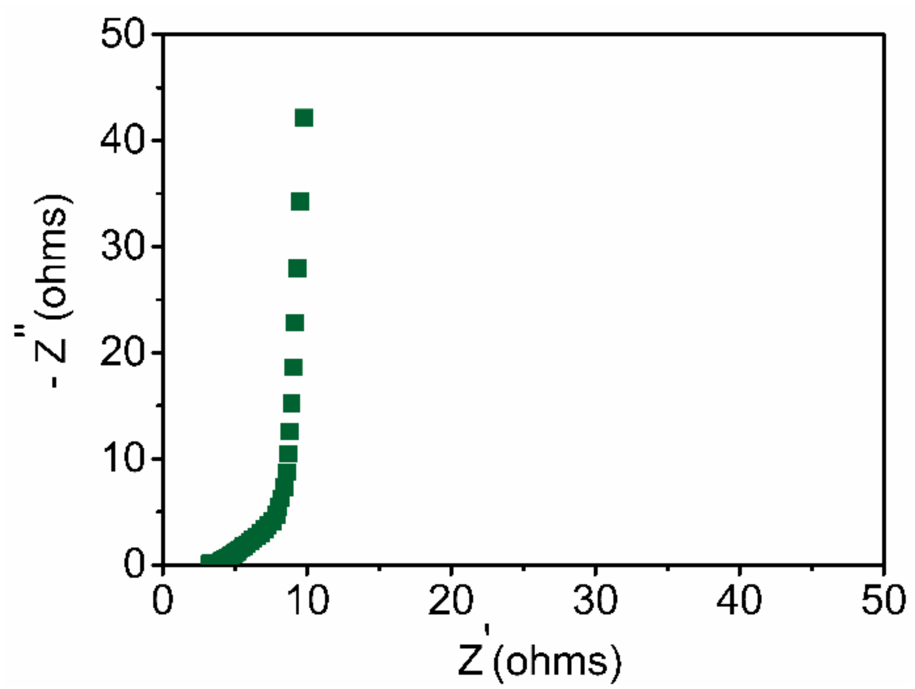

Figure S11. Nyquist plots of assembled HPC-4 based cell in two-electrode system in neat $EMIMBF_4$.

Table S1. N_2 adsorption–desorption isotherm parameters for waste CGs derived carbons.

**Samples**	**S_{BET} (m^2 g^{-1})**	**V_t (cm^3 g^{-1})**	**V_{t-DFT} (cm^3 g^{-1})**	**$V_{micropores-DFT}$ (cm^3 g^{-1})**	**$V_{meso/macropores-DFT}$ (cm^3 g^{-1})**
C-700-800	1218	0.59	0.57	0.46	0.11

Table S2. XPS parameters for coffee grounds derived carbons.

Samples	**C (%)**	**O (%)**	**N (%)**	**C_{1s}**			
				C-C (%)	C-O (%)	C=O (%)	O=C-O (%)
MC	93.2	5.6	1.2	75.0	15.3	5.3	4.4
HPC-2	92.7	7.2	0.1	61.2	29.1	3.3	6.4
HPC-4	91.4	8.6	0	55.7	32.1	4.1	8.1
HPC-6	88.1	11.9	0	46.9	37.5	7.3	8.3
